# Electrochemical and Optical Multi-Detection of *Escherichia coli* Through Magneto-Optic Nanoparticles: A Pencil-on-Paper Biosensor

**DOI:** 10.3390/bios14120603

**Published:** 2024-12-10

**Authors:** Furkan Soysaldı, Derya Dincyurek Ekici, Mehmet Çağrı Soylu, Evren Mutlugun

**Affiliations:** 1 Biological and Medical Diagnostic Sensors Laboratory (BioMeD Sensors Lab), Department of Biomedical Engineering, Erciyes University, Kayseri 38030, Türkiye; furkansoysaldi@nevsehir.edu.tr; 2 Department of Electronic and Automation, Vocational School, Nevsehir Haci Bektas Veli University, Nevsehir 50300, Türkiye; 3 Department of Nanotechnology Engineering, Abdullah Gul University, Kayseri 38039, Türkiye; derya.dincyurek@agu.edu.tr; 4 Department of Electrical-Electronics Engineering, Abdullah Gul University, Kayseri 38039, Türkiye; 5 UNAM–Institute of Materials Science and Nanotechnology, Bilkent University, Ankara 06800, Türkiye

**Keywords:** magneto-optic, biosensor, electrochemical impedance spectroscopy, quantum dots, Fe_2_O_3_@CdSe/ZnS

## Abstract

*Escherichia coli (E. coli)* detection suffers from slow analysis time and high costs, along with the need for specificity. While state-of-the-art electrochemical biosensors are cost-efficient and easy to implement, their sensitivity and analysis time still require improvement. In this work, we present a paper-based electrochemical biosensor utilizing magnetic core-shell Fe_2_O_3_@CdSe/ZnS quantum dots (MQDs) to achieve fast detection, low cost, and high sensitivity. Using electrochemical impedance spectroscopy (EIS) as the detection technique, the biosensor achieved a limit of detection of 2.7 × 10^2^ CFU/mL for *E. coli* bacteria across a concentration range of 10^2^–10^8^ CFU/mL, with a relative standard deviation (RSD) of 3.5781%. From an optical perspective, as *E. coli* concentration increased steadily from 10^4^ to 10^7^ CFU/mL, quantum dot fluorescence showed over 60% lifetime quenching. This hybrid biosensor thus provides rapid, highly sensitive *E. coli* detection with a fast analysis time of 30 min. This study, which combines the detection advantages of electrochemical and optical biosensor systems in a graphite-based paper sensor for the first time, has the potential to meet the needs of point-of-care applications. It is thought that future studies that will aim to examine the performance of the production-optimized, portable, graphite-based sensor system on real food samples, environmental samples, and especially medical clinical samples will be promising.

## 1. Introduction

Bacteria are everywhere in our lives, but pathogenic strains cause infectious diseases, epidemics and deaths in cases where the tracking and classification cannot be done accurately, quickly, easily, affordably and on-site. The development of rapid and sensitive methods for the effective detection of *E. coli* is important not only for patients but also for public health. Inadequate detection of *E. coli* results in prolonged treatment time and thus the spread and worsening of infectious diseases [1,2,3,4,5,6,7,8,9,10,11,12].

Conventional detection techniques currently utilized for *E. coli* detection consist of culture (plating and colony counting) methods, polymerase chain reaction (PCR) and enzyme-linked immunosorbent assay (ELISA) [2,3,7,11,12]. All of the traditional methods mentioned are too slow for the timely identification of an epidemic or bioterrorism attack response [13,14].

Nowadays, nanotechnology-supported optical measurement-based methods are in demand as they provide cheaper, safer, faster and more sensitive results [15]. Although fluorescent [16] (Limit of Detection (LOD): 8.3 × 10^3^ CFU/mL), colorimetric [17] (LOD: 10^4^ CFU/mL), surface-enhanced Raman spectroscopy [18] (LOD: 10^5^ CFU/mL), surface plasmon resonance [19] (LOD: 1.1 × 10^6^ CFU/mL) and chemiluminescence [20] (LOD: 10^3^ CFU/mL) methods are mostly preferred in optical detection, smartphone-connected, paper sensor-based sensors also come to the fore due to their advantages of low cost, portability and short detection time [21,22,23,24].

Another platform that stands out in bacterial detection is electrochemical sensors whose electrodes are modified with nanomaterials [25]. In particular, magnetic nanoparticle-based electrochemical sensors provide results with LODs as low as 1 × 10^2^ CFU/mL [26], 10 CFU/mL [27] and 2.84 × 10^3^ CFU/mL [28], respectively. In their review study, Štukovnik et al. (2023) stated that nanomaterial-supported impedimetric electrochemical biosensors have high selectivity, sensitivity and response time [29]. In a study combining the advantages of magnetic nanoparticles and impedimetric electrochemical sensors, the authors developed a sensitive, rapid and cost-effective assay for the detection of *Escherichia coli (E. coli)* in drinking water and apple juice [30]. The detection is based on electrical impedance spectroscopy measurements with screen-printed interdigitated electrodes combined with magnetic nanoparticles functionalized with the antimicrobial peptide melittin (MLT). With this approach, it was possible to detect *E. coli* concentrations of up to 1 CFU/mL in drinking water and 3.5 CFU/mL in apple juice with a linear range of 1–10^6^ CFU/mL in just 25 min without sample preparation. The disadvantages of the study are the high cost of sensor production, the need to process the impedance spectroscopy data with multidimensional projection techniques, and the time spent on the processes performed.

The popularity of quantum dots as nanomaterials in impedimetric biosensors has increased thanks to their distinctive qualities such as electrocatalytic activity, tunable size, good signal amplification and multiplex detection capability [31]. In the study reported by Zong et al. (2019), they utilized CdS quantum dots-encapsulated metal-organic frameworks as signal-amplifying tags and were able to detect *E. coli* at a sensitivity limit of 3 CFU/mL [32]. In the same study, they stated that the use of quantum dots has promising advantages in terms of sensitivity and selectivity in electrochemical immunosensor applications.

However, the literature is still lacking in a biosensor that can measure with high precision in a short time, which does not take long to prepare for the sensor, can enable optical and electrochemical measurement methods, and can do all these at low cost. Therefore, to overcome the transducer handicaps mentioned above with its fast and specific sensing capability, low cost and high sensitivity, our hybrid biosensor study combines a paper-based electrochemical biosensor and magnetic quantum dots (MQDs) in a novel approach. Our proposed biosensor has remarkable advantages as follows: (i) the sensing system is capable of taking measurements with high precision in 30 min, (ii) the sensor can be achieved using stock magnetic quantum dots within 90 min, (iii) the system utilizes low-cost materials such as paper and pencil, (iv) the system is capable of multi-detection with simultaneous electrochemical and optical sensing.

## 2. Materials and Methods

### 2.1. Materials

Cadmium oxide (CdO, 99.99%), zinc acetate (ZnAc, 99.99%), selenium (Se, 99.99% powder), sulfur (S, 99.998% trace metals basis), oleic acid (OA, 99.99% technical grade), 1-octadecene (1-ODE, 90% technical grade), trioctylphosphine (TOP, 97% technical grade), iron(0)pentacarbonyl (Fe(CO)_5_, >99.99% trace metals basis), oleylamine (OAm), Trimethylamine N-oxide (TMNO, 95%), N-hydroxysulfosuccinimide (Sulfo-NHS), N-(3-dimethylaminopropyl)-N′-ehylcarbodiimide hydrochloride (EDC), pyridine, Hexane (anhydrous, 95%), ethanol (EtOH, 99.8% absolute), acetone (Act, 99.5% absolute) and methanol (MeOH, absolute) were purchased from Sigma-Aldrich, *Escherichia coli* ATCC 25922 strain (0.5 McFarland), *Proteus mirabilis* and *Klebsiella pneumoniae* bacteria were obtained from Erciyes University Hospitals Central Laboratory, and *Escherichia coli* antibody ab25823 was purchased from Abcam Cambridge, UK. AIM-4300 Antenna Analyzer (5 kHz to 300 MHz) device was obtained from Array Solutions Sunnyvale, United States. 8B soft Staedtler pencil, Flormar brand nail polish, *Bacillus clausii* (Enterogermina 5 mL oral, Sanofi) and Neodymium Rectangle Magnet were obtained from Amazon Corp (Istanbul, Türkiye). All chemicals were used without further purification.

### 2.2. Fabrication of Graphite Sensors

**Figure 1:**
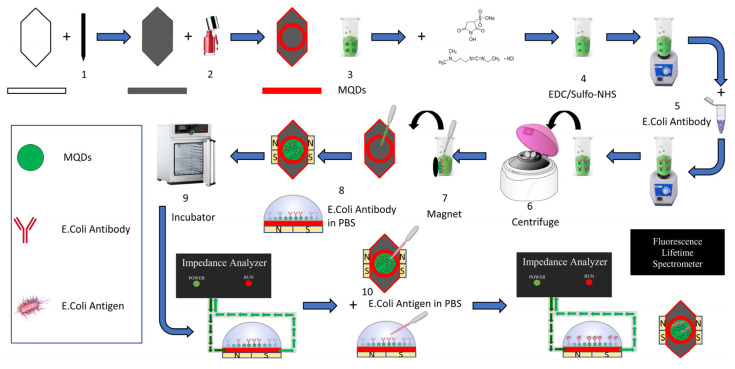
Biosensor fabrication, surface modification and bacteria detection schematics.

A 3.4 cm wide and 1.7 cm long sensor was created from standard A4 paper, weighing 80 g/m^2^. A surface area of 4.25 cm^2^ on the top of the sensor was painted with an 8B soft Staedtler pencil (Figure 1. Step 1). Thus, a graphite-coated surface was obtained. The coating was continued by taking measurements with a multimeter from time to time until the targeted conductivity value of 3 mS was achieved. In order to have a circular working area of 1.3 cm^2^ on this surface, approximately 1 cm^2^ circular area outside this working area was covered with commercial nail polish to create a hydrophobic area (Figure 1. Step 2). In addition, hydrophobic areas were created by completely covering the bottom and side of the sensor with nail polish. The fact that these individually produced sensors have the same conductivity value has been ensured by taking measurements with a multimeter unit.

### 2.3. Synthesis of Antibody-Modified CdSe/ZnS@Fe_2_O_3_ NPs

Synthesis of green-emitting QDs: For the synthesis of CdSe/ZnS quantum dots core/shellQDs, CdO (0.3 mmol) and zinc acetate (ZnAc, 4 mmol) were reacted with oleic acid (5 mL) in a three-neck flask. Evacuation is done until the vacuum level reaches 10^−3^ torr at 100 °C. 15 mL of 1-ODE is added to the solution temperature before it exceeds 100 °C. When the mixture is at 100 °C, the evacuation process is continued and 10^−3^ Torr vacuum level is attained. When the system reaches the desired vacuum pressure, the vacuum valve is closed and the N_2_ gas flow valve is opened. The solution temperature is slowly adjusted to 300 °C. Se (0.3 mmol), S (3 mmol), 2 mL TOP solution is prepared in a glove box and the mixture is mixed to a clear solution at 65 °C and 750 rpm stirring speed. This prepared solution is taken from the glove box and injected into the solution that has reached 300 °C, and the mixture is cooled immediately after 10 min of reaction time. After green CdSe/ZnS QD synthesis, washing is done (with hexane, acetone and methanol) to separate them from the excess ligands in the medium [33]. 

Synthesis of Yolk Iron Oxide Shell (Fe_2_O_3_) around the CdSe@ZnS Core: Vacuum-dried CdSe@ZnS nanoparticle sample (0.1491 g) was dispersed in a mixture of 20 mL ODE and OAm (the volume ratio of OAm/Fe(CO)_5_; 1.43) with sonication. The mixture is evacuated in a basket-type heater at 120 °C until the vacuum level reaches 10^−3^ Torr. When the vacuum value reaches the desired value, the vacuum valve is closed, the N_2_ gas is opened and the temperature is adjusted to 60 °C. When the temperature reaches 60 °C, 2 mL of Fe(CO)_5_ (Fe(CO)_5_/CdSe@ZnS by weight ratio; 0.7) solution is injected into the mixture. After the injection, the basket-type heater is withdrawn and the reaction is continued with the sonicator. The sonochemical reaction is kept for 30 min to allow ligand exchange to occur. After 30 min, a metallic Fe shell was formed around the CdSe@ZnS core. After the sonication process was completed, the solution temperature was adjusted to 100 °C in the basket heater, and the solution consisting of oxidizing agent TMNO (0.012 g) and ODE (5 mL) was added, and at this point, controlled oxidation of iron was achieved with the TMNO/ODE mixture. This resulted in the formation of a yellow Fe_2_O_3_ iron oxide shell enclosing CdSe@ZnS. Superior magnetic properties emerged after oxidation. Washing was performed to remove ligands with the formation of CdSe/ZnS@Fe_2_O_3_ [34]. Synthesis of MQDs is illustrated in Figure 1. Step 3.

The formed amine:(CdSe/ZnS@Fe_2_O_3_); 15 mg of Sulfo-NHS were dissolved in pyridine (5 mL). EDC (15 mg) in 0.25 mL of chloroform was added to the above solution. 40 mg of CdSe/ZnS@Fe_2_O_3_ NPs was added, and then the mixture was shaken for 2 h. The formed amine:(CdSe/ZnS@Fe_2_O_3_) NPs were precipitated by the addition of hexane, separated by an external magnet, and washed with ethanol and H_2_O [35]. In this way, the sensor surface was functionalized as amine reactive.

Synthesis of antibody-(CdSe/ZnS@Fe_2_O_3_) NPs; 5 mg amine group modified magnetic quantum data were weighed and 180 µL pH (7.4) sodium phosphate buffer was added and sonicated to disperse the particles. In order to provide antibody modification, 20 µL amin active of *E. coli* antibody with 10^15^ concentration was added to this prepared solution. This mixture was sonicated for 30 min and then the reaction was completed in an orbital mixer for 45 min. Bacteria were made reactive with MQDs, EDC/Sulfo-NHS and antibodies as illustrated in Figure 1 Steps 4 and 5. The mixture is centrifuged for about 1–2 min until it settles (Figure 1. Step 6). The amine active antibodies were linked via a stable amide bond. At the end of the procedure, washing was done 3 times with a phosphate buffer to remove unbound antibodies.

### 2.4. Preparation of the Sensor Surface

The prepared antibody-(CdSe/ZnS@Fe_2_O_3_) NPs were held by an external magnet, 200 µL were dropped onto the detection surface of graphite-coated (Figure 1. Steps 7 and 8) and polished paper-based biosensors and left to dry for 15 min at 40 °C in the incubator device (Figure 1. Step 9). After the drying process, the sensors are ready for analysis with impedance and quantum efficiency.

### 2.5. Characterization Techniques for CdSe/ZnS@Fe_2_O_3_ NPs

Transmission electron microscopy (TecnaiG2-F30 TEM, FEI, Hillsboro, OR, USA) was utilized to analyze the morphology of yolk-shell CdSe/ZnS@Fe_2_O_3_ nanoparticles. A 2 μL droplet of the CdSe/ZnS@Fe_2_O_3_ solution, dissolved in hexane, was placed onto a copper grid and subsequently air-dried. The TEM employed a field emission electron source operating at 200 keV for imaging. Energy-dispersive X-ray (EDX) analysis was carried out on a Zeiss Gemini 300 model device (Oberkochen, Germany). Infrared (IR) spectra were collected on Thermo Scientific Nicolet 6700 FTIR spectrophotometer (Waltham, MA, USA).

### 2.6. Detection of E. coli Bacteria by Prepared Sensors

A magnet was placed under the modified MNPs (bare Fe_2_O_3_ as magnetic nanoparticle) and MQDs (magnetic Fe_2_O_3_ coated CdSe/ZnS) on the surface of the prepared multifunctional sensors to better contact the Carbon atoms on the graphite-covered surface by utilizing magnetism, and then they were connected to the probes of the electrochemical impedance spectroscopy device (AIM4300, Array Solutions, Sunnyvale, TX, USA) for impedance measurement. 150 µL of phosphate buffer was added on it and the first measurement was taken only when the antibody was bound. Then the buffer solution was drawn again and *E. coli* bacteria (concentration of 10^8^, 10^7^, 10^6^, 10^5^, 10^4^, 10^3^, 10^2^ CFU/mL was added in 150 µL phosphate buffer (Figure 1. Step 10). Impedance measurements were taken at different times (t = 0, 10, 20, 30 min). After 30 min, the sensor surface was washed with phosphate buffer and unbound bacteria were removed from the sensor. After the washing process, impedance measurement was taken again when 150 µL of phosphate buffer solution on the sensor surface and the data were recorded.

In order to measure the bacterial specificity of the prepared device, in the above experimental procedure, measurements were made with *E. coli*, *Proteus mirabilis*, *Klebsiella pneumoniae* and *Alkalihalobacillus clausii* (*Bacillus clausii*) bacteria at a concentration of 10^6^ CFU/mL in 150 µL. 

In addition, test and control experiments with 10 repetitions each were performed to test the stability of the system. For this, the first solution containing *E. coli*, *Proteus mirabilis*, *Klebsiella pneumoniae* and *Alkalihalobacillus clausii* bacteria at a concentration of 10^6^ CFU/mL in 150 µL, and the second solution containing *Proteus mirabilis*, *Klebsiella pneumoniae* and *Alkalihalobacillus clausii* bacteria at a concentration of 10^6^ CFU/mL in 150 µL were prepared. And experiments were conducted as test and control respectively. In our research, all experiments were carried out in at least 3 repetitions.

Time-correlated single photon counting is a powerful technique to monitor the excitonic interactions on the emissive MQDs. We utilize this tool to simultaneously monitor the effect of the interaction between the bacteria and MQDs. After the process was finished, the changes in the irradiation kinetics of the bacteria-bound and non-bacteria-bound sensors were analyzed in a real-time device for each sample using PicoQuant FluoTime 200 (Berlin, Germany). 

The raw data from all stages of the experiments were taken one by one and transferred to the data processing software, these data were manipulated with the nonlinear curve fit (parabole) process, as much as 1000 data, and fitting was applied. For the data that could not reach 1000 data, extrapolation was performed. R_s_ calculation was made by considering the points where the data intersects the x-axis.

### 2.7. QCM Characterization 

To clean the gold surface of a 5 MHz AT-cut quartz crystal (Biolin Scientific–QSX 301, Gothenburg, Sweden), it was placed in a 10-fold de-ionized water (DIW) (Erciyes University Environmental Engineering, Kayseri, Türkiye) diluted solution of piranha (1.5 mL sulfuric acid (1120801000, Merck, Istanbul, Türkiye), 0.5 mL hydrogen peroxide (Merck) for at least 2 min. Then the sensor was washed sequentially with ethyl alcohol (Merck, 99.9%) and distilled water. The cleaned gold surface was exposed to a solution of 2.5 mM cysteamine (Merck) in de-ionized water for 12 h. Frequency shifts in the QCM crystal were monitored with an impedance analyzer (Array Solutions, AIM-4300, Sunnyvale, TX, USA). Amine-reactivated MQDs at a concentration of 5 mg/mL in 1× Phosphate Buffered Saline (PBS, 18912014, Thermo Fisher, Istanbul, Türkiye) solution was applied to the QCM sensor for 30 min by means of a peristaltic pump (Longer Pump, BT100-2J, Istanbul, Türkiye).

#### Detection System

After each measurement step, PBS washing was performed with a peristaltic pump for 5 min. Anti-*E. coli* antibody at a concentration of 10^13^ Ab/mL in PBS was applied to the sensor surface for 30 min. After the surface was blocked for non-specific binding by applying 1% BSA solution in PBS to the surface, the stability of the sensor in the negative control was followed for 30 min. In the last step, detection was completed by applying *E. coli* bacteria with a concentration of 10^7^ CFU/mL in 1% BSA + PBS to the sensor surface.

## 3. Results

**Figure 2:**
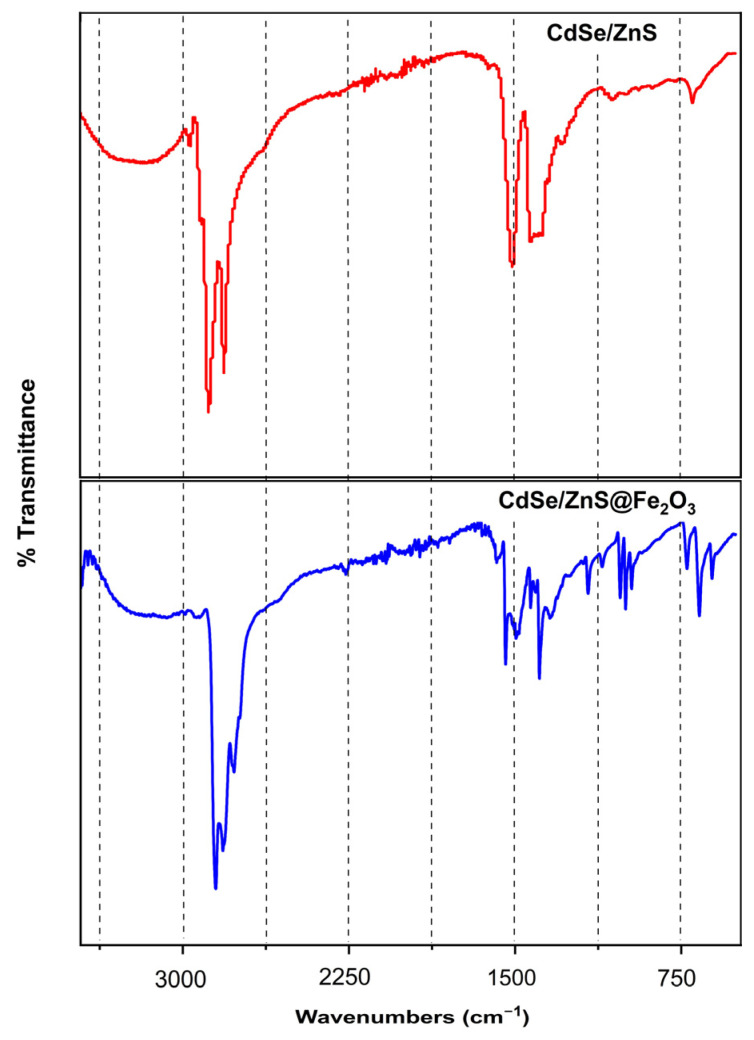
The FTIR analyses of CdSe/ZnS and CdSe/ZnS@Fe_2_O_3_ nanoparticles.

The FTIR spectra of the synthesized CdSe/ZnS@Fe_2_O_3_ nanocomposite and the bare CdSe/ZnS are shown in Figure 2.

The characteristic peaks of CdSe/ZnS are around 3004 and 2900 cm^−1^ corresponding to the stretching vibrations of O-H and C-O bands, respectively. Two sharp peaks around 1540 and 1454 cm^−1^ were observed due to the stress vibrations of the thiol coating.

Additionally, for the CdSe/ZnS@Fe_2_O_3_ nanocomposite, the peaks around 694 cm^−1^ in FTIR show the characteristic vibration peak of Fe-O and these peaks indicate the presence of maghemite [36,37].

**Figure 3:**
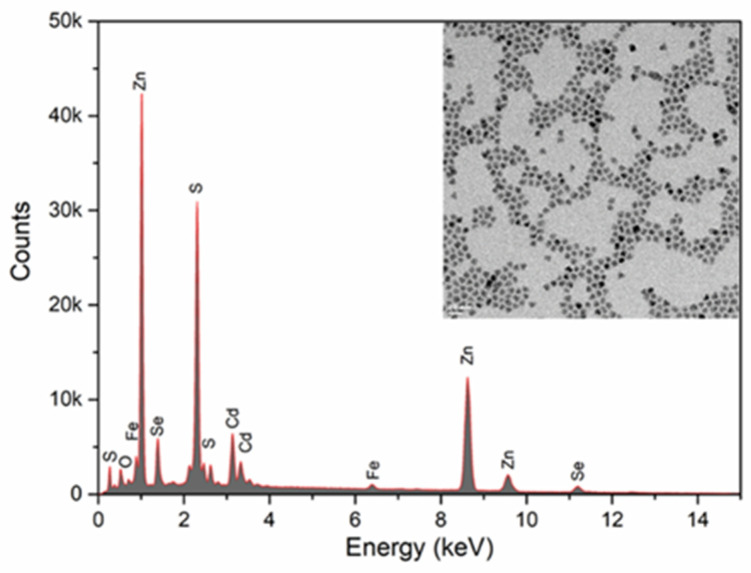
The Energy-dispersive X-ray spectroscopy of CdSe/ZnS@Fe_2_O_3_ nanoparticles and TEM analyses.

Figure 3 reveals the elemental stoichiometry of the nanoparticles by Energy Dispersive X-ray analysis (EDX) (Zeiss, Gemini 300, (Oberkochen, Germany)) along with the transmission microscopy image. The observed peaks are consistent with the chemicals used. Since iron is in the form of a thin layer of chelate, it should appear in low amounts and the results are consistent with the amount of material used.

Table 1 shows the elemental analysis of the synthesized magnetic quantum dots.

The equivalent circuit model of the impedimetric biosensor is shown in Graphic Abstract in the impedance analysis measurements made at frequencies ranging from 5 kHz to 5 MHz [38]. 

**Figure 4:**
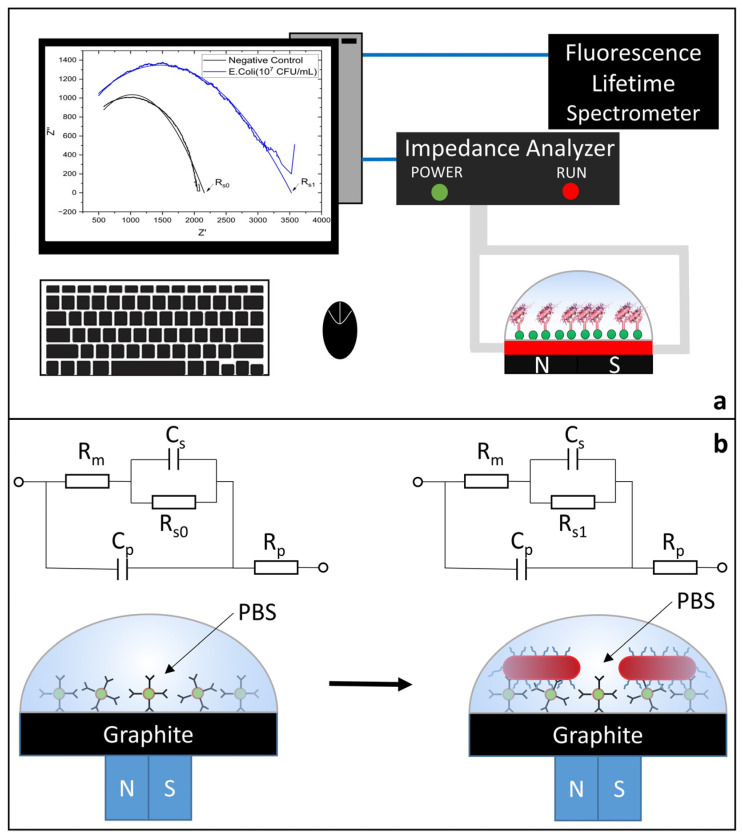
(**a**) Detection System including the PC interface, impedance analyzer and fluorescence lifetime spectrometer (not to scale). (**b**) The equivalent circuit model.

In the equivalent circuit model (Figure 4a,b), R_m_ represents the impedance of the biosensor consisting of medium solution resistance, R_s_ electrode-solution interface resistance, C_s_ electrode-solution interface capacitance, C_p_ the parasitic capacitance and R_p_ parasitic resistance [38].

**Figure 5:**
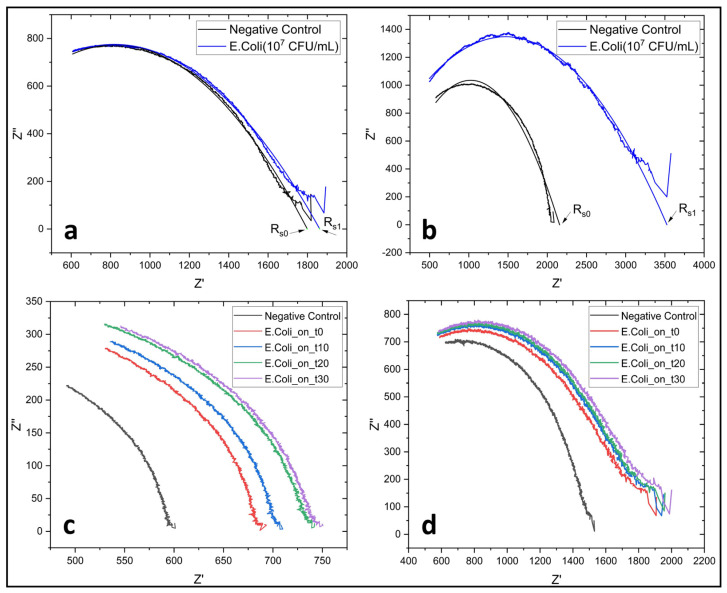
(**a**) Fitted data and raw data of 5 mg MNP with 10^7^ CFU/mL *E. coli* data. (**b**) Fitted data and raw data of 5 mg MQD with 10^7^ CFU/mL *E. coli* MQD data. (**c**) Time dependent saturation of bacteria on 5 mg MNP. (**d**) Time dependent saturation of bacteria on 5 mg MQD.

The study conducted in Figure 5 aims to compare MNP and MQD in terms of sensitivity based on the percentage change in the R_s_ value and to determine the time it takes for the sensor surface to reach saturation at a specific target concentration. Given at Figure 5a,b, it is seen that R_s_ value also increases with bacteria bound as a result of antibody-antigen interaction. The R_s0_ value in the related graphs shows the electrode-solution interface resistance in PBS when there are modified nanoparticles on the graphite sensor surface. R_s1_, on the other hand, shows the electrode-solution interface resistance of the sensor in PBS after the washing process after the antibodies on the nanoparticle bind to the bacteria. R_s0_ and R_s1_ are used to show the percent change in relative electrode-solution interface resistance. It can be seen that the difference between R_s0_ and R_s1_ is less in Figure 5a and more in Figure 5b. This difference shows that MQD produces more sensitive results than MNP. Looking at Figure 5c,d, it is seen that the R_s_ values, which are the basic criteria for bacteria detection, reach saturation in 30 min in both MNP and MQD sensors.

Experiments were started using 1 mg MNP in order to detect 10^7^ CFU/mL *E. coli* bacteria, as seen in Figure S1. 18.57026 (±10.4499) %ΔR_s_ change was obtained. In the later part of the experiment, 5 mg MNP was tested and 24.94076 (±0.76582) %ΔR_s_ results were obtained. At this point, approximately 40% more change was achieved. Afterwards, 1 mg MQD was used. The change 37.72521 (±4.3315) %ΔR_s_ was obtained. 1 mg MQD produced approximately 50% more change than 5 mg MNP. Afterwards, 5 mg MQD was used and the change 52.434 (±6.7400) %ΔR_s_ was obtained. In this case, similar to the literature review, approximately 40% more change was obtained compared to 1 mg MQD and approximately 110% more change than 5 mg MNP [39,40]. With this comparison, it was decided to use 5 mg MQD in the further experiments.

To elucidate the role of quantum dots in enhancing sensor performance, we compared the electrochemical response of graphene + Fe_2_O_3_ + antibody electrodes with graphene + Fe_2_O_3_ + quantum dots (CdSe/ZnS) + antibody electrodes. Impedance spectroscopy revealed a ~52% relative change in ΔR for the quantum-dot-incorporated electrode compared to ~25% for the Fe_2_O_3_ –only electrode for 5 mg dry nanoparticle samples. This enhancement is attributed to the superior electrocatalytic activity and increased surface area provided by quantum dots, enabling more efficient charge transfer and greater antibody binding capacity. Furthermore, the quantum dots enable optical detection, offering dual detection capabilities that are absent in the Fe_2_O_3_ –only electrode. These results underscore the critical role of quantum dots in achieving higher sensitivity, specificity, and multimodal detection in the proposed hybrid biosensor (Figure S1).

**Figure 6:**
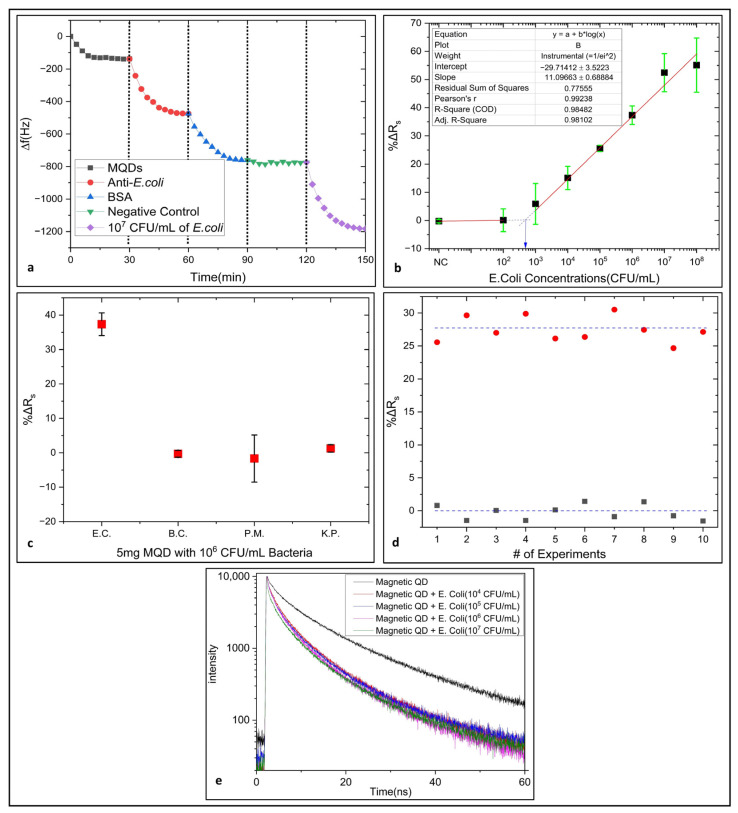
(**a**) Frequency shift responses were obtained for each step in the detection of *E. coli* ATCC 25922 bacteria by surface-modified QCM sensor with magnetic quantum dots (MQDs). (**b**) Dose-Response. (**c**) Specificity of the 5 mg MQD system. (**d**) Stability Test. (**e**) The average lifetime of the quantum dots for different concentrations of *E. coli* bound on them.

The functionality of the graphite sensor surface functionalization methodology used in the study has been confirmed by the QCM, which is valid in biosensor applications with the data made on the QCM and showing all the steps (Figure 6a).

We demonstrated that the MQDs on the graphite sensor are functional by using a QCM sensor. By functionalizing the MQDs, which we immobilized on the QCM gold surface with a covalent bond, using the same cross-linking agents and receptor in our study, we demonstrated on the generally accepted [41,42,43] mass sensitive QCM sensor that we can detect [44,45] bacteria with this functionalization method.

Using 5 mg magnetic quantum dots (MQDs), with *E. coli* bacteria at negative control (NC), 10^2^, 10^3^, 10^4^, 10^5^, 10^6^, 10^7^ and 10^8^ CFU/mL concentrations, −0.2422 (±0.58), 0.0914 (±4.0532), 5.8769 (±7.2423), 15.0806 (±4.1235), 25.5584 (±1.1232), 37.3312 (±3.2752), 52.434 (±6.74) and 55.10 (±9.6033) ΔR_s_ (%) changes were detected, respectively.

To determine the limit of detection and linear dynamic range of the biosensor, the curve whose equation is given between the concentrations of 10^3^ CFU/mL and 10^8^ CFU/mL, with a linear relationship observed in the graph in Figure 6b, was fitted. The intersection point of the curve fitted and extended between negative control and 10^2^ CFU/mL concentration and the curve reported above is seen. This point is the limit of detection point and its value is determined as 2.7 *×* 10^2^ CFU/mL [46].

In light of these results, it can be stated that the hybrid sensor platform is a highly sensitive and wide-ranging sensor, with a detection limit of 2.7 *×* 10^2^ CFU/mL and a linear dynamic range of 10^3^ CFU/mL to 10^8^ CFU/mL, for detecting *E. coli*.

An *E. coli* concentration of ≥10^5^ CFU/mL in asymptomatic individuals, or *E. coli* concentration of ≥10^2^ CFU/mL in symptomatic patients, is typically considered as a UTI [47].

The handmade paper-based graphite sensor, which has been variously modified to detect *E. coli* bacteria, was specification tested with other bacteria that cause urinary infections in which *E. coli* was detected. These bacteria are gram-negative like *E. coli*, *Proteus mirabilis*, *Klebsiella pneumoniae* and also gram-positive *Alkalihalobacillus clausii* (*Bacillus clausii*). All bacteria were tested to a concentration of 10^6^ CFU/mL. As seen in Figure 6c, the system whose antibody of *E. coli* bacteria is modified on the sensor surface does not respond to bacteria other than *E. coli*. These values are respectively: for *E. coli* (EC) 37.3312 (±3.2752) %ΔR_s_, for *Bacillus clausii* (BC) −0.2944 (±0.9840) %ΔR_s_, for *Proteus mirabilis* (PM) −1.6675 (±6.8444) %ΔR_s_ and for *Klebsiella pneumoniae* (KP) 1.2737 (±1.0803) %ΔR_s_. In this way, it shows that it is quite specific [48].

As seen in Figure 6d, 10 consecutive stability control experiments were performed with the system to detect *E. coli* bacteria in the control medium containing *Proteus mirabilis*, *Klebsiella pneumoniae* and *Alkalihalobacillus clausii* bacteria at a concentration of 10^6^ CFU/mL in 150 µL, except for *E. coli* bacteria. As a result of these experiments, a change of −0.2422 (±0.58) %ΔR_s_ was obtained.

Then as seen in Figure 6c, 10 successive stability test experiments were performed with the system to detect *E. coli* bacteria in the test medium containing *E. coli* bacteria, *Proteus mirabilis*, *Klebsiella pneumoniae* and *Alkalihalobacillus clausii* bacteria at a concentration of 10^6^ CFU/mL in 150 µL. As a result of these experiments, a change of 27.4529 (±0.9823) %ΔR_s_ was obtained. 

Looking at the dose response graph in Figure 6b, it is seen that the change at 10^6^ CFU/mL concentration is 37.3312 (±3.2752) %ΔR_s_. But in the stability test, the change was 27.4529 (±0.9823) %ΔR_s_. In other words, there is a decrease in change of approximately 27%. The reason for this was the presence of bacteria with similar characteristics in the detection environment and the interference of these bacteria.

The repeatability of each experimental set was calculated by the following equation:

Relative Standard Deviation (RSD)(%) = σ/μ.100

σ represents the standard deviation of the concentrations measured in each experimental set and μ represents the mean value of the concentrations measured in each experimental set.

In Figure 6d, black dots are control experiments and red dots are test experiments. As a result of the stability repeatability study, the relative standard deviation (RSD) (%) value of the sensor was found to be 3.5781, according to the formula mentioned above.

To inaugurate a novel path for bacterial detection and ensure the tangible perception of bacteria on the quantum dot’s surface, we employed time-correlated single photon counting measurements on magnetic quantum dot specimens. By modulating the bacterial concentration on the MQD, facilitated by the excitonic interactions between MQDs and bacteria, we methodically discerned accelerated emission decays in the samples. This acceleration signifies the extent of interaction, even discernible at exceedingly low bacterial concentrations.

Figure 6e presents the time-correlated single photon count decays of the MQD complex. Here, the same hierarchical procedure was utilized with the impedance measurements. 

The bare lifetime of the quantum dots, before any bound *E. coli* on the surface have been measured as 8.59 ns. The lifetimes of the magnetic quantum dots were extracted as 3.37, 3.72, 3.92 and 4.66 ns, respectively, at 10^7^, 10^6^, 10^5^ and 10^4^ CFU/mL *E. coli* concentrations dropped on the sensor surface. As the concentration of the *E. coli* increased steadily from 10^4^ to 10^7^ CFU/mL, the average lifetime of the quantum dots decreased down to 3.37 ns demonstrating over 60% lifetime quenching.

With standard A4 paper, 8B pencil and nail polish, a biosensor that is very cost-effective, simple, fast, portable, sensitive, specific, stable and accurate measurements were obtained. CdSe/ZnS quantum dots with Fe_2_O_3_ magnetic shell, surface modified with amine groups, antibodies recognizing *E. coli* bacteria attached to the detection point on the surface of graphite-coated paper biosensors were immobilized with the help of a magnet, and both an electrochemical and optical sensor were obtained. In this way, all the advantages desired to be obtained from an electrochemical biosensor and an optical biosensor were combined [49,50,51].

While no additional external cross-check experiments were conducted, the developed hybrid biosensor was validated through its dual-mode detection approach (electrochemical impedance and optical fluorescence lifetime), specificity testing against non-*E. coli* bacteria, and repeated stability analyses. The consistency of results across these independent tests provides robust confirmation of the method’s accuracy. Furthermore, the sensor’s performance metrics, such as sensitivity, limit of detection, and specificity, are in agreement with previously reported studies, further corroborating the validity of the results. These validations demonstrate that the hybrid biosensor is a reliable tool for accurate *E. coli* detection.

## 4. Discussion

With the biosensor, whose cost is very low and sensor fabrication is very easy and fast, both electrochemical and optical measurements can be achieved at the same time. This is a measure to increase sensitivity and accuracy. Apart from this, using the nanoparticles with a diameter of around 13 nm, have a reasonably high active area, gives faster results in interaction with bacteria, and creates a sensor that can detect even small concentrations. The fabricated sensor and MQDs and their surface modifications were confirmed by electrochemical impedance spectroscopy (EIS) and fluorescence time-correlated single photon counting method (TCSPC) Through this approach, the sensor production methods are very simple, the sensor is produced in 10 min and the functionalized magnetic quantum dots to detect *E. coli* can be made ready on the sensor surface within 90 min. Furthermore, *E. coli* detection allows on-site measurements with electrochemical and optical detection systems on the sensor surface within 30 min. Different studies related to this subject have been investigated and our comparisons are given in Table 2.

The UTI threshold for asymptomatic individuals is 10^5^ CFU/mL and for symptomatic patients is 10^2^ CFU/mL and when the table above is examined, our proposed hybrid biosensor is quite sensitive in detecting such low concentrations with a detection limit of 2.7 × 10^2^ CFU/mL [47,59,60]. Additionally, the sensitivity of our biosensor can be further increased with methods such as recombinant antibody technology [61,62], sandwich detection methodology [63,64], and accessory proteins such as protein A and protein G [35,65] that allow the correct orientation of the receptor.

In the studies summarized in the table, sensor preparation takes quite a long time. In our study, the sensor is made ready for detection in a short time of 90 min, and the detection system allows measurement by mutual verification with two different detection platforms. Another advantage of the dual measurement system in our study is that it makes an accurate and sensitive quantitative measurement with impedimetric measurement [66,67,68]. In addition, MQDs that emit at different wavelengths can be specified for different strains and bacteria and a solution that would allow the detection of different strains and bacteria at the same time can be proposed.

The novelty of this study lies in the development of a hybrid biosensor that integrates paper-based electrochemical detection with optical sensing, utilizing magnetic quantum dots (MQDs) as the core sensing element. This dual-mode approach, which is not commonly reported in the literature, allows simultaneous detection using two complementary techniques, enhancing both sensitivity and accuracy. The MQDs, comprising CdSe/ZnS quantum dots with a Fe_2_O_3_ magnetic shell, provide a high surface-to-volume ratio for effective antibody immobilization and dual magnetic/optical properties. These features enable rapid sensor preparation (90 min), a low detection limit of 2.7 × 10^2^ CFU/mL, and specificity in distinguishing *E. coli* from other bacterial species. Furthermore, the use of low-cost and easily accessible materials such as standard A4 paper, pencil graphite, and nail polish underscores the practicality of this sensor for point-of-care diagnostics. This work bridges the gap between high-performance detection systems and cost-effective, portable platforms, presenting a significant advancement in biosensing technologies.

## 5. Conclusions

In our study, a paper-based electrochemical biosensor and a hybrid biosensor produced from Fe_2_O_3_@CdSe/ZnS nanoparticles with both optical and magnetic properties will provide an advantage for such studies due to its fast and specific detection capability, low cost and high sensitivity. The designed biosensor specifically recognizes and quickly analyzes *E. coli* bacteria. One of the most important features of this biosensor, which has a very low cost, is that it can perform optical analysis while performing on-site electrochemical analysis. Thanks to this bidirectional analysis, the analysis accuracy of the biosensor is ensured. The fact that the materials with both magnetic and optical properties used on the surface of the biosensor become specific with the ease of surface modification, and their size is around 13 nm, has provided more active areas. Thus, even at low concentrations (2.7 × 10^2^ CFU/mL), the rapid detection (30 min) of a wide linear dynamic range of 10^3^ CFU/mL to 10^8^ CFU/mL *E. coli* bacteria provided significant advantages. As a result of the stability study, each with 10 repetitions, the relative standard deviation (RSD) (%) value of the sensor was found to be 3.5781. The change in the average lifetime of the materials of magnetic quantum dots with the specific binding of *E. coli* bacteria to the surface was investigated. The bare lifetime of the quantum dots before any *E. coli* on the surface is bound has been measured to be 8.59 ns. As the *E. coli* concentration increased (from 10^4^ to 10^7^ CFU/mL), the average lifetime of the quantum dots decreased to 3.37, showing a lifetime decrease of over 60%.

It is anticipated that the proof of concept demonstration of such an optically enhanced electrochemical sensor with a fast, sensitive and specific detection base will open up new possibilities for the nanotechnology-supported bacterial detection systems and bio-sensing platforms.

The results of this study demonstrate significant advancements in biosensor technology by integrating optical and electrochemical detection modalities into a single platform. This hybrid approach, facilitated by magnetic quantum dots (MQDs), provides enhanced sensitivity (limit of detection: 2.7 × 10^2^ CFU/mL) and specificity, addressing limitations of single-mode sensors reported in the literature. Compared to traditional methods, our sensor reduces preparation and detection times while maintaining high performance. The use of MQDs not only amplifies signal responses but also ensures efficient and stable immobilization of antibodies, leading to reliable detection in complex biological matrices. Importantly, the cost-effectiveness and portability of the paper-based design make it suitable for resource-limited settings, emphasizing its potential for public health and environmental monitoring. Future developments could expand this dual-detection approach to other pathogens, incorporating multiplexed detection capabilities for comprehensive diagnostic solutions.

## Figures and Tables

**Table 1:** Elemental analysis of CdSe/ZnS@Fe_2_O_3._

Element	Weight%	Atomic%
O K	2.24	7.36
S K	22.16	36.4
Cd L	9.62	4.51
Fe K	1.04	0.98
Zn K	53.61	43.2
Se K	11.33	7.56

**Table 2:** Comparison of Studies Detection of *E. coli* by Detection Method.

Method	Sensor Preparation Time (min)	Detection Method (Electrochemical (EC)/Optical (O))	Limit of Detection (CFU/mL)	Detection Time (min)	Reference
Multiple amplification strategy via 3D DNA walker	750	EC	7	Not specified	[2]
Immunosensor	180	EC	30	60	[3]
Aptasensor based on Urease catalysis amplification strategy	120	EC	12	5	[7]
Immunosensor	210	EC	2	2	[8]
Personal Glucometer (PGM) Immunoassay	4260	EC	1.83 × 10^2^	90	[9]
Nonenzymatic immunoassay	3600	EC	4.5 × 10^2^	40	[10]
Aptasensor	Not specified	EC	10	5	[11]
pH sensitive nanofiber	Not specified	EC	10^2^	60	[12]
Sandwich type immunosensor	180	EC	3	120	[32]
SsDNA	180	EC	10^2^	60	[52]
Aptamer	150	EC	80	30	[53]
Aptasensor	1790	EC	17	35	[54]
Aptasensor	Not specified	EC	2	40	[55]
Immunosensor	260	EC	4	60	[56]
Carbon Quantum Dot(CQD)-Based Label-Free Fluorescent	755	O	185	Not specified	[57]
Fluorescent CQDs SsDNA	870	O	60	60	[58]
This work	90	EC-O	2.7 × 10^2^	30	

## Data Availability

Data is available upon request.

## Supplementary Materials

The following supporting information can be downloaded at: https://www.mdpi.com/article/10.3390/bios14120603/s1, Figure S1: Comparison of the methods of detecting 10^7^ CFU/mL *E. coli* bacteria with different amounts of MNP and MQD.
