# Supplementary material for: Electrochemical and Optical Multi-Detection of Escherichia coli Through Magneto-Optic Nanoparticles: A Pencil-on-Paper Biosensor
Source: Biosensors (Basel). 2024 Dec 10;14(12):603. doi: 10.3390/bios14120603 (PMC11674942; doi:10.3390/bios14120603)
Supplement: Supplementary file 1 [file biosensors-14-00603-s001.zip › biosensors-3297374-supplementary.pdf]

Electrochemical and Optical Multi-detection of Escherichia coli through Magneto-optic Nanoparticles: A Pencil-on-paper Biosensor

Furkan Soysaldi[a,b], Derya Dincyurek Ekici[c], Mehmet Çağrı Soylu[*a] and Evren Mutlugun[*d,e]

[a]*Biological and Medical Diagnostic Sensors Laboratory (BioMeD Sensors Lab), Department of Biomedical Engineering, Erciyes University, Kayseri 38030, Türkiye*

[b]*Department of Electronic and Automation, Vocational School, Nevsehir Haci Bektas Veli University, Nevsehir, 50300, Türkiye*

[c]*Department of Nanotechnology Engineering, Abdullah Gul University, Kayseri, 38039, Türkiye*

[d]*Department of Electrical-Electronics Engineering, Abdullah Gul University, Kayseri, 38039, Türkiye*

[e]*UNAM – Institute of Materials Science and Nanotechnology, Bilkent University, Ankara, 06800, Türkiye*

Corresponding Authors: mcsoylu@erciyes.edu.tr, evren.mutlugun@agu.edu.tr

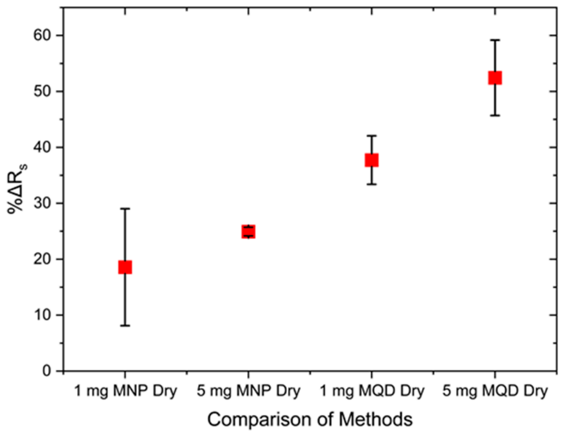

Figure S1. Comparison of the methods of detecting 10^7 CFU/mL *E.coli* bacteria with different amounts of MNP and MQD.

Experiments were started using 1 mg MNP in order to detect 10^7 CFU/mL *E. coli* bacteria, as seen in Figure S1. 18.57026 (±10.4499) %ΔR_s change was obtained. In the later part of the experiment, 5 mg MNP was tested and 24.94076 (±0.76582) %ΔR_s results were obtained. At this point, approximately 40% more change was achieved. Afterwards, 1 mg MQD was used. The change 37.72521 (±4.3315) %ΔR_s was obtained. 1 mg MQD produced approximately 50% more change than 5 mg MNP. Afterwards, 5 mg MQD was used and the change 52.434 (±6.7400) %ΔR_s was obtained. In this case, similar to the literature review, approximately 40% more change was obtained compared to 1 mg MQD and approximately 110% more change than 5 mg MNP [1,2]. With this method comparison, it was decided to use 5 mg MQD in the experiments.

REFERENCES

(1) Schrattenecker, J. D.; Heer, R.; Melnik, E.; Maier, T.; Fafilek, G.; Hainberger, R. Hexaammineruthenium (II)/(III) as alternative redox-probe to Hexacyanoferrat (II)/(III) for stable impedimetric biosensing with gold electrodes. *Biosensors and Bioelectronics* **2019**, *127*, 25-30.

(2) Karuppiah, S.; Mishra, N. C.; Tsai, W.-C.; Liao, W.-S.; Chou, C.-F. Ultrasensitive and low-cost paper-based graphene oxide nanobiosensor for monitoring water-borne bacterial contamination. *ACS sensors* **2021**, *6* (9), 3214-3223.
